# Fellow-Workers and the Roll of Honour

**Published:** 1915-04-03

**Authors:** 


### ROUND THE WORLD.

[The Editor invites Early Information and Contributions Marked "Fellow-Workers."]

### Fellow-Workers and the Roll of Honour.

The recent fierce fighting on the British Front has taken further heavy toll of the R.A.M.C. So many brave medical officers can ill be spared just now, when all available medical and surgical skill is required for the large armies about to take the field. Amongst those who have lately fallen must be mentioned Captain H. V. B. Byatt, Lieut. W. T. McCurry, Lieut. J. R. Waddy, and Lieut. D. C. Turnbull.

Captain Harry Vivian Byatt Byatt fell mortally wounded on March 11, being in action with the Rifle Brigade (2nd Battalion). He entered the R.A.M.C. soon after becoming an L.R.C.P., M.R.C.S. in 1907, being then in his twenty-sixth year, and was promoted four years ago.

Lieut. Derwent Christopher Turnbull lost his life through trying to get a wounded officer out of the danger zone on March 10, on which occasion he was badly hit, succumbing a few days later. A Sheffield student, he took the qualifying degrees of M.B., B.Ch. there last year, and, joining the R.A.M.C. in December, was appointed to the Cheshire Regiment (1st Battalion). He was only twenty-four years old.

Sir Charles Pardey Lukis, K.C.S.I., the much respected Director-General of the Indian Medical Service, is to be greatly sympathised with on the loss of his eldest son, Lieut. Theodore Stewart Lukis, R.A.M.C. A brilliant student of St. Bartholomew's, where he held the responsible posts of demonstrator of physiology, house physician, and ophthalmic house surgeon, Lukis won the gold medal at the M.D. Lond. three years ago, and more recently became an M.R.C.P. Lond. Keenly interested in social work, his influence in Hoxton was such that he was able to call nearly a hundred young men to the flag when the emergency came, whilst he himself accepted a commission. In addition to his work at "Bart.'s" he had been house physician to the Great Ormond Street Hospital for Sick Children.

Lieutenant Walter Tennyson McCurry was likewise one of that eager band of brilliant young men who answered the call as soon as the war broke out, and who have passed away from life and activity with a quickness that seems doubly appalling to their fellow-workers and friends left behind. The international crisis found McCurry a senior but unqualified student of the Irish schools. Without hesitation he entered for the qualifying examination of the Dublin Apothecaries' Hall then about to be held, and as an "L.A.H. Dub." became attached to the 15th Field Ambulance. Through the struggle round Mons, St. Quentin, and near Le Cateau, then serving through the victory on the Marne, he came home for a short well-earned rest at Christmas, and after returning to the Continent was sent to the Norfolk Regiment. His early death is much lamented at Queen's College, Belfast, as also amongst those who knew him at the Dublin hospitals.

Lieutenant John Raymond Waddy, who qualified L.R.C.P., M.R.C.S. in 1912, was a student of Cambridge and King's College Hospital. After qualifying he settled in practice at Taunton, but joined the R.A.M.C. as one of the additional lieutenants appointed on the outbreak of war. His gallantry had been rewarded by the Military Cross some little time before a stray shot put an end to his career. Inspiriting stories have been told of his bravery in attending our wounded under heavy fire, particularly during the heavy fighting just before Christmas.

Lieutenant Alexander Seabrooke, lately senior obstetric resident at Guy's, is one of the students of that hospital who have just joined the R.A.M.C. Proceeding to Guy's from Cambridge, Seabrooke soon exercised a notable influence amongst his fellows, who looked up to him as a sportsman and a gentleman. After qualifying L.R.C.P., M.R.C.S. in 1912, he took the M.B., B.C. Cantab., and then held every possible resident appointment, excepting that of ophthalmic house surgeon, before joining the Army. Lieutenant Seabrooke takes with him into his new sphere of activity a host of good wishes from his late *confrères* in London and Cambridge.

Fleet-Surgeon P. W. Bassett-Smith, R.N., has been carrying out important experiments at the Royal Naval College, Greenwich, in conjunction with Sir Watson Cheyne and Mr. Arthur Edmunds, F.R.C.S., with the object of determining the best anti-microbic dressing to apply to infected wounds. Reporting on his results to the *British Medical Journal* he draws attention to the importance of salicylic acid as a disinfectant of tissues attacked by virulent anaërobic germs. Mr. Bassett-Smith, who qualified in 1882 and became an M.R.C.P. Lond. two years ago, is one of the most distinguished officers of the Royal Naval Medical Service. A Middlesex Hospital man, he has for many years given special attention to bacteriological problems and tropical medicine, subjects on which he lectures for the naval authorities.

Dr. Gilbert Lowell Webb, who has been appointed surgeon to the military hospital at Fort Mahon, is a Cambridge and St. Thomas's man, who settled in practice at Bexhill-on-Sea. After taking his qualifying degree at Cambridge in 1906 he held various hospital posts, including those of resident medical officer to the British Lying-in Hospital, as also house physician and house surgeon to the West London Hospital. A keen ambulance worker and examiner to the St. John Ambulance Association, Dr. Webb's new work lies in connection with the Tenth French Army.

Lieutenant Henry Colpoys Gloster, another hero of the Neuve Chapelle action, killed on March 12, was a medical student who had passed his first M.B. at Cambridge, and was proceeding to his more advanced studies when war was declared. He then obtained a commission in the Gordon Highlanders, and was promoted to lieutenant after a few weeks' training. Going to the Front soon after, he carried out meritorious work in the trenches during the winter. Lieutenant Gloster was the only son of Dr. James Gloster, of Kensington.

